# *Brucella ceti* Infection in Striped Dolphins from Italian Seas: Associated Lesions and Epidemiological Data

**DOI:** 10.3390/pathogens12081034

**Published:** 2023-08-13

**Authors:** Carla Grattarola, Antonio Petrella, Giuseppe Lucifora, Gabriella Di Francesco, Fabio Di Nocera, Antonio Pintore, Cristiano Cocumelli, Giuliana Terracciano, Antonio Battisti, Ludovica Di Renzo, Donatella Farina, Cristina Esmeralda Di Francesco, Maria Ines Crescio, Simona Zoppi, Alessandro Dondo, Barbara Iulini, Katia Varello, Walter Mignone, Maria Goria, Virginia Mattioda, Federica Giorda, Giovanni Di Guardo, Anna Janowicz, Manuela Tittarelli, Fabrizio De Massis, Cristina Casalone, Giuliano Garofolo

**Affiliations:** 1 Istituto Zooprofilattico Sperimentale del Piemonte, Liguria e Valle d’Aosta, 10154 Torino, Italy; mariaines.crescio@izsto.it (M.I.C.); simona.zoppi@izsto.it (S.Z.); alessandro.dondo@izsto.it (A.D.); barbara.iulini@izsto.it (B.I.); katia.varello@izsto.it (K.V.); waltermignone.wm@gmail.com (W.M.); maria.goria@izsto.it (M.G.); virginia.mattioda@izsto.it (V.M.); federica.giorda@izsto.it (F.G.); cristina.casalone@izsto.it (C.C.); 2 Istituto Zooprofilattico Sperimentale della Puglia e della Basilicata, 71121 Foggia, Italy; antonio.petrella@izspb.it (A.P.); donatella.farina@izspb.it (D.F.); 3 Istituto Zooprofilattico Sperimentale del Mezzogiorno, 89852 Vibo Valentia, Italy; giuseppe.lucifora@izsmportici.it; 4 Istituto Zooprofilattico Sperimentale dell’Abruzzo e del Molise, 64100 Teramo, Italy; g.difrancesco@izs.it (G.D.F.); l.direnzo@izs.it (L.D.R.); 5 Istituto Zooprofilattico Sperimentale del Mezzogiorno, 80055 Portici, Italy; fabio.dinocera@izsmportici.it; 6 Istituto Zooprofilattico Sperimentale della Sardegna, 07100 Sassari, Italy; antonio.pintore@izs-sardegna.it; 7 Istituto Zooprofilattico del Lazio e della Toscana, 00178 Roma, Italy; cristiano.cocumelli@izslt.it (C.C.); antonio.battisti@izslt.it (A.B.); 8 Istituto Zooprofilattico del Lazio e della Toscana, 56312 Pisa, Italy; giuliana.terracciano@izslt.it; 9 Faculty of Veterinary Medicine, University of Teramo, 64100 Teramo, Italy; cedifrancesco@unite.it (C.E.D.F.); gdiguardo@unite.it (G.D.G.); 10 National and OIE Reference Laboratory for Brucellosis, Istituto Zooprofilattico Sperimentale dell’Abruzzo e del Molise, 64100 Teramo, Italy; a.janowicz@izs.it (A.J.); m.tittarelli@izs.it (M.T.); f.demassis@izs.it (F.D.M.)

**Keywords:** *Brucella ceti*, marine mammals, neuropathology, cetaceans, Italy, sequence type

## Abstract

*Brucella ceti* infections have been increasingly reported in cetaceans. In this study, we analyzed all cases of *B. ceti* infection detected in striped dolphins stranded along the Italian coastline between 2012 and 2021 (*N* = 24). We focused on the pathogenic role of *B. ceti* through detailed pathological studies, and ad hoc microbiological, biomolecular, and serological investigations, coupled with a comparative genomic analysis of the strains. Neurobrucellosis was observed in 20 animals. The primary histopathologic features included non-suppurative meningoencephalitis (*N* = 9), meningitis (*N* = 6), and meningoencephalomyelitis (*N* = 5), which was also associated with typical lesions in other tissues (*N* = 8). Co-infections were detected in more than half of the cases, mostly involving Cetacean Morbillivirus (CeMV). The 24 *B. ceti* isolates were assigned primarily to sequence type 26 (ST26) (*N* = 21) and, in a few cases, ST49 (*N* = 3). The multilocus sequence typing (cgMLST) based on whole genome sequencing (WGS) data showed that strains from Italy clustered into four genetically distinct clades. Plotting these clades onto a geographic map suggests a link between their phylogeny and the topographical distribution. These results support the role of *B. ceti* as a primary neurotropic pathogen for striped dolphins and highlight the utility of WGS data in understanding the evolution of this emerging pathogen.

## 1. Introduction

The genus *Brucella* contains an increasing number of species, some of which are of relevant to public health and economic concerns in many areas of the world [1,2,3].

*Brucella* spp. infections were first described in pinnipeds and cetaceans from California and Scotland in the early 1990s [4,5], and have been since reported in several wild marine mammal species all over the world. Since 2007, isolates of *Brucella* spp. from marine mammals have been further classified into two species, *B. ceti* and *B. pinnipedialis*, preferentially associated with cetaceans and pinnipeds, respectively [6]. *Brucella ceti* has been isolated from dolphins, whales, porpoises, and some pinnipeds [6,7,8,9,10,11].

Pathological changes associated with *B. ceti* infection in cetaceans are well documented, and include reproductive tract inflammation (endometritis, orchitis), abortions, placentitis, mastitis, pneumonia, myocarditis, pericarditis, osteoarthritis, spinal discospondylitis, subcutaneous abscesses, hepatic, splenic, and lymph node necrosis, alongside macrophage infiltration in the liver and spleen, and meningoencephalomyelitis [8,9,12,13,14,15,16,17,18,19,20,21,22,23,24,25,26,27,28,29,30].

Neurobrucellosis represents a common cause of stranding for cetaceans, being associated with disorientation, uncoordinated lateral swimming, buoyancy disturbances, and death [30], while it has not been recorded in bovine, caprine, ovine, swine, or canine hosts [31]. Moreover, based on the infection patterns documented at the central nervous system (CNS) level in striped dolphins (*Stenella coeruleoalba*), which resemble those seen in human patients [32], a specific susceptibility has been suggested for this species [9,12,16,17,18,31,33,34,35,36].

*B. ceti*, similarly to other *Brucella* species, seems to replicate inside host macrophages and trophoblasts [9,16,17], but mechanisms of pathogenesis, virulence, and host affinity, including the cell receptor(s) involved in the host’s CNS invasion, have not yet been fully understood [9,31,37,38].

The localization of *B. ceti* in lungworms and cestodes raises the possibility that they may serve as vectors for the transmission of the infection [9,20], although the role of metazoan parasites in the eco-epidemiology and pathogenesis of brucellosis in cetaceans is still unclear.

According to Multi Locus VNTR (Variable Number of Tandem Repeats) Analysis (MLVA), *B. ceti* strains can be divided into two major clusters and three sub-clusters [39,40]. Multi Locus Sequence Typing (MLST) has been used to identify 13 sequence types (STs) to date, as reported in the public PubMLST repository (https://pubmlst.org/brucella/, accessed on 27 April 2023).

Only a few cases of infection by *B. ceti* sequence type (ST) 27 have been documented in humans [7,9,30], and therefore the zoonotic potential of marine *Brucella* species remains ill-defined.

Although *B. ceti* infection is of increasing concern among free-ranging cetaceans in most oceans across the world [9,29,41] there is limited information about isolates from Mediterranean Sea cetaceans. *B. ceti* was first isolated in 2009 from a striped dolphin stranded along the Spanish Catalonian coast [21]. In 2012, *B. ceti* infection was documented in four other cetaceans: in one striped dolphin and one bottlenose dolphin (*Tursiops truncatus*) found beached along the same Spanish Catalonian coastline [21], and in two striped dolphins stranded along the Tyrrhenian and Adriatic Sea Italian coastlines [18,42]. Based on MLST investigations, all *B. ceti* strains isolated in these cases belonged to ST26 [21,42].

In Italy, a coinfection involving *Brucella* spp. was then molecularly confirmed in 2015, in a striped dolphin stranded along the Ligurian coastline, and was associated with related pathological changes in the brain, blubber, liver, and spleen [23]. Moreover, a *B. ceti* ST27 strain, previously found only in Pacific Ocean waters [2,9], was isolated from several lymph nodes of one bottlenose dolphin in the Croatian part of the northern Adriatic Sea, thus representing the first evidence of the spread of the strain in Mediterranean as well as in European waters [43,44]. The first survey of *B. ceti* infection in eight striped dolphins stranded along the coast of Italy from 2012 to 2018 [36] showed the presence of ST26 strains in all cases, with an apparently higher occurrence of the infection along the Ionian coastline.

To gain proper insight into the epidemiology and the pathological features of *B. ceti* infection in cetaceans found stranded along the Italian coastline, we analyzed all cases of *B. ceti* infection detected by microbial isolation—the gold standard diagnostic test—in striped dolphins stranded between 2012 and 2021 (*N* = 24). We focused on the pathogenic role of the microorganism as well as on the genetic make-up of the strains involved. All strains were subjected to a comparative genomic analysis using whole genome sequencing (WGS) to characterize the fine phylogenetic relationships and infer phylogeographic distribution in dolphin populations of the Italian seas. Moreover, we aimed at determining whether different *B. ceti* STs are linked to specific *B. ceti*-associated lesions, stranding areas, age classes, and the “CeMV infection *status*” of the animals involved.

## 2. Materials and Methods

### 2.1. Dolphins and Samples Included in the Study

We investigated specimens of 24 striped dolphins found stranded and lifeless on the coast of Italy between 2012 and 2021, all of which were positive for the isolation of *B. ceti* from one or more tissues during routine pathological and cause-of-death assessment. This assessment was performed at the diagnostic public laboratories belonging to the network of Istituti Zooprofilattici Sperimentali (II.ZZ.SS.), coordinated by the National Reference Centre for Diagnostic Investigations on Stranded Marine Mammals (C.Re.Di.Ma.), which was officially established in 2014 by the Italian Ministry of Health.

**Figure 1:**
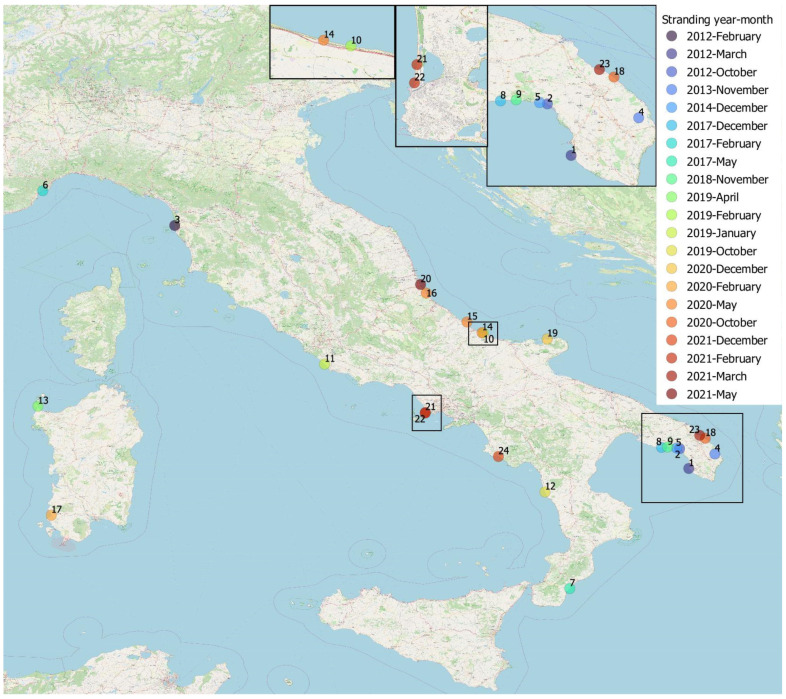
Stranding sites of striped dolphins infected by *B. ceti* under study, Italy, 2012–2021, by month and year of stranding. Geographical mapping was obtained by QGIS software using the geographical coordinates found from the strandings (source monitoring of cetacean strandings on Italian coasts, http://mammiferimarini.unipv.it/index_en.php, accessed on 18 May 2023). Each dot represents a stranding and is identified by the Case Id number of Supplementary Table S1. The Italy map was used under a CC BY-SA copyright from OpenStreetMap contributors (https://www.openstreetmap.org/copyright/en, accessed on 18 May 2023).

The geographical distribution of the stranding events for the animals under study is recorded in Figure 1.

### 2.2. Post-Mortem Examination

The stranded animals were examined and necropsied according to standard guidelines, depending on the carcasses’ preservation status [45,46]. Only a limited sampling was carried out on the carcass of Case 4.

Each specimen was labeled with the IZS identification code, alongside the code assigned by the Banca Dati Spiaggiamenti (BDS) (http://mammiferimarini.unipv.it, accessed on 18 May 2023). We reorganized all the concerned cases in chronological order, from Case 1 to Case 24.

Stranding (type, location, date) and life history data (species, sex, estimated age class) were recorded. The stranding locations of animals under investigation were reviewed to identify the associated sea sector (http://mammiferimarini.unipv.it, accessedon 18 May 2023).

During the necropsy, the decomposition condition category (DCC) and the nutritional condition category (NCC) were evaluated [46].

The carcass DCC at the time of necropsy was classified as code 1 (extremely fresh carcass, just dead), code 2 (fresh), code 3 (moderate decomposition), code 4 (advanced decomposition), or code 5 (mummified or skeletal remains) [46].

The carcass NCC was assessed and classified as good, moderate, or poor [46].

The age class was established, based on total body length (TBL) [47], in three estimated age classes (newborns/calves, juveniles, and adults), with the final differentiation between juveniles and adults being made based on gonad maturation [48,49].

Macroscopical findings of all cases were recorded, and the gastric chambers were opened to evaluate pathological changes and their content. The presence of helminths was estimated by macroscopic and microscopic examination of tissues. Endoparasites were preserved in 70% alcohol for microscopic identification according to established morphological characteristics [50,51].

During necropsy, tissue samples from all the major organs and lesions were collected and subsampled: one was kept frozen at −20 °C for microbiological investigations and one was kept frozen at −80 °C for biomolecular analyses, with the remaining one being preserved in 10% buffered formalin for histological and immunohistochemical (IHC) investigations.

The brain was cut into two halves, and one half was fixed in 10% neutral buffered formalin and the other was split into two separate portions, one frozen at −20 °C and the other at −80 °C. The spinal cord from some dolphins was also organized in a similar way on a metameric basis (cervical, thoracic, lumbo-sacral regions).

Whenever available, cerebrospinal fluid (CSF), blood serum, and aqueous humor (HA) were collected and frozen at −20 °C for serological investigations. Selected tissues and/or fluids (CSF) were collected for microbiological, biomolecular, and serological investigations focused on *Brucella* infection diagnosis.

### 2.3. Diagnostic Investigations

Table 1 shows an overview of the conducted analyses per case, arranged in chronological order.

For histological investigations, CNS samples included the cerebrum in all animals. In some cases, cerebellum, medulla oblongata, and spinal cord were also sampled. Coronal sections from different regions (telencephalon, diencephalon, mesencephalon, pons, cerebellum, medulla, and spinal cord) [52] as well as samples from all major organs were fixed in 10% neutral buffered formalin, embedded in paraffin, sectioned at 4 μm and finally stained with hematoxylin and eosin (HE) for light microscopy examination.

As ancillary diagnostic investigations and to deepen the pathological findings observed or the positivity to biomolecular investigations, immunohistochemistry (IHC) for Morbillivirus was performed on tissue sections of Cases 6, 12, 21, and 22 including the brain, as well as on the urinary bladder from Case 6, using a monoclonal anti-canine distemper virus (CDV) antibody (VMRD, Pullman, WA, USA) [53].

*Toxoplasma gondii* IHC was carried out on the brain tissues of Cases 6, 12, and 21 using a polyclonal serum of caprine origin (VMRD, Pullman, WA, USA) [53].

Molecular detection of relevant pathogens such as CeMV [54] and *T. gondii* [55] was carried out in each animal under study, with CNS tissue samples systematically tested for both pathogens, except case 19, in which the CNS was tested only for *T. gondii*, and cases 10, 14, 15, and 16, in which the CNS was tested only for Morbillivirus. Moreover, additional available tissues, consisting of lung, liver, spleen, lymph nodes, heart, kidney, skeletal muscle, urinary bladder, intestine, skin, and skin ulcer, were tested for Morbillivirus, while liver, skeletal muscle, spleen, lymph nodes, heart, thymus, and intestine tissue samples were tested for *T. gondii*.

Furthermore, the presence of Herpesvirus (HV) was investigated by PCR [56] in CNS samples and additional tissues, including spleen, lymph nodes, skin, lung, liver, kidney, skin ulcer, and tongue ulcer of Cases 6, 11, 17, 21, 22, and 24.

Serological investigations aimed at assessing the occurrence of anti-Morbillivirus and anti-*T. gondii* antibodies were also performed in 6 animals (Cases 3, 6, 7, 12, 14, 15) [53] and, specifically, in the blood serum, CSF, and aqueous humor of Case 6, as well as in the blood serum and CSF of Cases 12 and 14, and in the blood serum of Cases 3, 7 and 15.

### 2.4. Investigations Focused on Brucella Infection Diagnosis

#### 2.4.1. *Brucella* Isolation and Identification

In-depth microbiological investigations targeting *Brucella* spp. were performed in CNS samples of all animals and, except for Cases 2, 4, and 5, also in the other tissues available, including spleen, lymph nodes, lung, liver, heart, pancreas, kidney, urinary bladder, ovarium, uterus, testicle, mammary gland, muscle, cerebrospinal fluid (CSF), and a lungworm.

The frozen CNS of Case 7 was used for bacterial isolation subsequent to the observation of microscopic lesions suggestive of neurobrucellosis discovered during the histopathological analysis performed retrospectively on the CNS from this dolphin. Likewise, frozen tissues available for Cases 13 and 17 were investigated after microscopic lesions suggestive of neurobrucellosis had been found during routine investigations.

The *Brucella* spp. isolation and identification procedures were performed in accordance with the technique described in the OIE Manual of Diagnostic Tests and Vaccines [57], using both selective and non-selective solid media and enrichment broths to enhance the chance of isolating the microorganism (except for tissues other than CNS of Case 7).

For Cases 1, 2, 4, and 5, Farrell’s and Columbia blood agar media were used, while for Case 3, and for Cases 6 and 7, Farrell’s and Modified Thayer Martin’s solid media, and a combination of Farrell’s and CITA media were used, respectively. Considering all the other cases, modified Thayer Martin and CITA media were used. The solid media were incubated at 37 °C, aerobically and in a microaerophilic atmosphere containing 5% CO_2_, for at least 10 days. An enhancement step was carried out in *Brucella* enrichment broth, supplemented with fetal horse serum, and modified *Brucella* selective supplement, and incubated at 37 °C in a microaerophilic atmosphere containing 5% CO_2_. For Case 3 we used trypticase-soy broth supplemented with amphotericin B (1 mg/mL) and vancomycin (20 mg/mL), and for Cases 8, 9, 18, 19, and 23 we also added Thayer Martin broth. Enrichment cultures were subcultured weekly (six subcultures) on selective solid media described above. Suggestive colonies (circular, convex, shiny, 1–2 mm in diameter after 48–72 h) were seeded onto blood agar medium and incubated for a further 2 days before re-examination. When *Brucella* spp. was suspected based on Gram’s staining [58], the colonies were tested for catalase, oxidase, and urease activities [58]. Motility and slide agglutination tests with *Brucella* anti-A and anti-M antisera were also performed for Cases 1, 2, 4, 5, 8, 9, 18, 19, and 23, together with nitrate reduction, H_2_S production, and growth in the presence of CO_2_ for Cases 6, 7, 12, 13, 17, 21, 22, and 24 [58].

For DNA extraction, all *B. ceti* isolates were subcultured on *Brucella* medium agar base (BAB; Oxoid, Hampshire, UK) and incubated in a 5–10% CO_2_ atmosphere at 37 °C for 48 h to assess the purity of cultures and the absence of dissociation. Bacterial DNA was extracted from single colonies using the Maxwell1 16 Tissue DNA Purification Kit by means of Maxwell1 16 Instrument (Promega, Madison, WI, USA), or the High Pure DNA Template Preparation kit (Roche Diagnostics, Mannheim, Germany) according to the manufactures’ instructions. All strains isolated from the striped dolphins under study were identified as *B. ceti* using the PCR-RFLP method [59] and then subjected to genomic analysis at the National and OIE Reference Laboratory for Brucellosis, Istituto Zooprofilattico Sperimentale dell’Abruzzo e del Molise, Teramo, Italy.

#### 2.4.2. Molecular Detection of *Brucella* spp. from Tissues

The molecular detection of *Brucella* spp. was performed from available tissues of Cases 3, 6, 7, 8, 9, 10, 11, 12, 13, 14, 15, 16, 17, 20, 21, 22, and 24, including the CNS of all animals, except for Case 12, as well as from spleen, lymph nodes, laryngeal tonsil, lung, liver, heart, pancreas, kidney, urinary bladder, ovarium, uterus, testicle, mammary gland, muscle, tongue, skin, cerebrospinal fluid (CSF), and a lungworm.

Frozen tissue samples of Cases 3, 6, 7, 11, 12, 13, 17, 21, and 22 were tested by PCR to detect *Brucella* spp. using hemi-nested PCR targeting of an outer membrane protein gene of *B. abortus* [60].

Samples of Cases 10, 14, 15, 16, 20, and 24 were analyzed by RT-PCR for *Brucella* spp. [61].

Samples of Cases 8 and 9 were tested using multiplex PCR assay for the detection of *Brucella* spp. [62,63].

#### 2.4.3. Serological Tests for Brucellosis

Serological investigations aimed at assessing the presence of anti-smooth *Brucella* spp. antibodies were performed on the blood serum of eleven animals (Cases 6, 7, 9, 10, 12, 14, 15, 18, 19, 20, and 23), by rapid serum agglutination (Rose Bengal plate test, RBT), using RBT antigen produced from *B. abortus* strain S99 [16,53].

#### 2.4.4. Whole Genome Sequencing and Bioinformatics

Each strain submitted for genomic analysis was labeled with a corresponding ID number (ID strain 10759, 28753, 1207793, 31957, 2780, 3838, 17753, 1259, 25153, 2785, 19005477, 578, 6980, 39595, 46223, 66805, 26087, 255877, 19759, 260676, 277922, 277892, 226242, 28356), as listed in Supplementary Table S1. Total genomic DNA of the 16 samples from the new cases studied was sequenced with the Illumina NextSeq 500 instrument. Total genomic DNA was quantified using Qubit DNA HS assay (Thermo Fisher Scientific Inc., Waltham, MA, United States) and the sequencing libraries were generated using a Nextera XT Library Preparation Kit (Illumina, St. Diego, CA, United States). The libraries were sequenced in 300 cycles using NextSeq 500/550 Mid Output Reagent Cartridge v2, according to manufacturer’s instructions, thereby generating 150 bp paired end reads. Following automatic demultiplexing and adapter removal, the quality was assessed using FastQC v0.11.5 [64]. The reads were then quality trimmed using Trimmomatic v 0.36 [65] with the following quality parameters—Leading: 25; Trailing: 25; Slidingwindow: 20:25. Genome scaffolds were assembled using SPAdes version 3.11.1 (using parameters–k 21, 33, 55, 77; –careful) [66]. The quality of the scaffolds was assessed with QUAST v 4.3 [67]. Sequence reads were deposited in Sequence Read Archive (SRA) database under NCBI Bioproject PRJNA623338. Twenty-four assembled genomes from strains included in this study and from the previous study by Garofolo (2020) were imported into Ridom SeqSphere+ software, version 6.0.2 [68] where multilocus sequence typing (MLST) and core genome MLST (cgMLST) were performed. Briefly, MLST was assigned using a nine locus MLST (MLST-9) scheme available at https://pubmlst.org/brucella/ (accessed on 18 May 2023) and accessed through Ridom SeqSphere+. The cgMLST profiles were assigned using a template composed of 2067 core genes in Ridom SeqSphere+. The UPGMA tree was constructed by pairwise comparison of the cgMLST profiles with missing values ignored.

### 2.5. Cases/Pathological Evaluation

A hypothesis on the cause of death for all animals included in this study, except for Case 4, was formulated considering the biological and epidemiological data, coupled with the macroscopic and microscopic findings, alongside the results of all diagnostic investigations. The causes of death were categorized into causes of natural origin (pathologies of infectious origin, neonatal/perinatal pathologies, traumatic intra-interspecific interactions, senescence/aging, etc.) and anthropogenic (interaction with fishing, ship collisions, etc.) according to available bibliographic references [69,70] and recently elaborated diagnostic frameworks (“Evidence Based Diagnostic Assessment Framework for Cetacean Necropsies on Marine Debris Ingestion and Common Data Collection” (Ascobans 2020), Life DELFI harmonized protocols and frameworks (https://lifedelfi.eu/wp-content/uploads/2021/04/A3_Framework_Fishery_interaction 1.pdf, accessed on 18 May 2023). The gross and microscopic findings from all the *B. ceti*-microbiologically positive striped dolphins were reviewed. In data analysis, a lesion was categorized as *B. ceti*-associated (primary pathogen or co-pathogen) if associated with the simultaneous isolation of the pathogen in culture and/or with a positive result in the PCR assay, as well as with pathological features consistent with those previously described in the literature [8,9,12,16,17,18,22,25,26,27,28,29,30,31].

The features of *B. ceti* infection in all the animals under investigation were additionally evaluated to assess the microbial agent’s pathogenic role as the most probable cause(s) of stranding and/or death (Supplementary Table S1).

### 2.6. Statistical Analyses

Firstly, we performed a univariate analysis with the non-parametric Wilcoxon–Mann–Whitney test to detect differences between ST26 and ST49 in the distribution of either individual information (sex, age class) or the presence of *B. ceti*-associated lesions in the available organs (CNS, reproductive system, lymph nodes, spleen, liver, heart, lung, and mammary gland), or the presence of CeMV coinfection. Statistical significance was achieved when the *p*-value was <0.05. Multivariate analysis was performed by means of multi-level mixed effect logistic models, including the strain as the dependent variable, age class and the occurrence of *B. ceti*-associated pathologic changes in the available organs alongside the presence of CeMV coinfection as independent variables, and the individual as a random effect. All statistical analyses were performed using STATA 17.0.

## 3. Results

### 3.1. Postmortem Pathological and Diagnostic Laboratory Investigations

#### 3.1.1. Individual Data and Gastric Contents

Individual data (history, sex, age class, sexual maturity, DCC, NCC, stranding location, latitude and longitude coordinates, sea sector, date), along with gastric contents, gross and microscopic pathologic findings, *Brucella* analytical data (isolation, PCR, RBT), ancillary diagnostic test results (Morbillivirus/*T. gondii*/Herpesvirus), co-infections, and hypotheses about the cause(s) of death and the pathogenic role of *B. ceti* infection for each of the *B. ceti*-infected striped dolphins found stranded along the coast of Italy are summarized in Supplementary Table S1.

Excluding Case 4, in which sex could not be determined, females represented a higher proportion of stranded striped dolphins (15/23; 65.2%) than males (8/23; 34.8%).

Considering the age, adults (14/22; 63.6%) were overrepresented compared with juveniles (8/22; 36.4%). In Cases 4 and 23, the age class could not be estimated. Most of the animals were fresh (DCC 2) (18/22; 81.8%), with a few individuals exhibiting a moderate decomposition (DCC 3). For two animals (Cases 4 and 5), we were no able to estimate DCC.

Excluding Cases 4 and 23, in which NCC could not be estimated, the cetaceans under study showed different body conditions: poor (12/22; 54.5%), moderate (5/22; 22.7%), and good (5/22; 22.7%). Considering the stranding location, different sea sectors were represented: Ionian Sea (*N* = 6), Central Adriatic Sea (*N* = 6), Southern Adriatic Sea (*N* = 3), Central Tyrrhenian Sea (*N* = 3), Southern Tyrrhenian Sea (*N* = 2), Sardinian Sea (*N* = 2), and Ligurian Sea (*N* = 2).

With reference to gastric contents, except for Cases 2, 4, and 5, for which no data were available, most of the animal showed absence of ingesta (20/21; 95,2%), with evidence of a recent meal having been found in one animal only (Case 20) (1/21; 4.8%).

#### 3.1.2. Gross and Microscopic Findings

Postmortem examination and histopathological investigations were performed on 23 out of the 24 striped dolphins with positive culture for *B. ceti* (Case 4 was not included), and histopathologic evaluation of brain tissue samples was conducted in all the 23 cases.

A wide variety of gross and microscopic findings were observed in 21 out of the 23 investigated cetaceans (Supplementary Table S1).

##### General Gross Findings

The main gross findings included parasitization by *Phyllobotrium (delphini* and/or *Monorygma grimaldi* plerocercoids (20/23; 86.9%), hyperemic meninges and/or brain (17/23; 73.9%), a parasitic bronchopneumonia (13/23; 56.5%), a lymphadenomegaly (12/23; 52.1%), and a nodular gastritis by *Pholeter gastrophilus* (7/23; 30.4%) (Supplementary Table S1).

##### Gross *Brucella ceti*-Associated Findings

The main gross *Brucella ceti*-associated lesions included the hyperemic meninges and/or brain (17/23; 73.9%), alongside reproductive tract inflammation (endometritis) (4/23; 17.3%). Another prominent macroscopic change was represented by an increased volume or hemorrhagic CSF appearance (3/8; 37.5%) (Table 2; Supplementary Table S1).

##### Microscopic Findings Associated with *Brucella ceti* Infection (CNS)

Neurobrucellosis was observed in most of the animals investigated (20/23) (Table 2), and the main microscopic *Brucella ceti*-associated lesions were represented by non-suppurative meningoencephalitis, detected in 9/20 individuals (45%), non-suppurative meningitis in 6/20 (30%), and non-suppurative meningoencephalomyelitis in 5/20 (25%) (Supplementary Table S1).

In particular, the *B. ceti*-associated lesions detected in the CNS of bacteriologically and/or molecularly positive animals are shown in Table 3, along with the pathologic changes observed in CNS regions other than the cerebrum, whenever present, and/or lesions related to other pathogens in case of coinfection.

**Figure 2:**
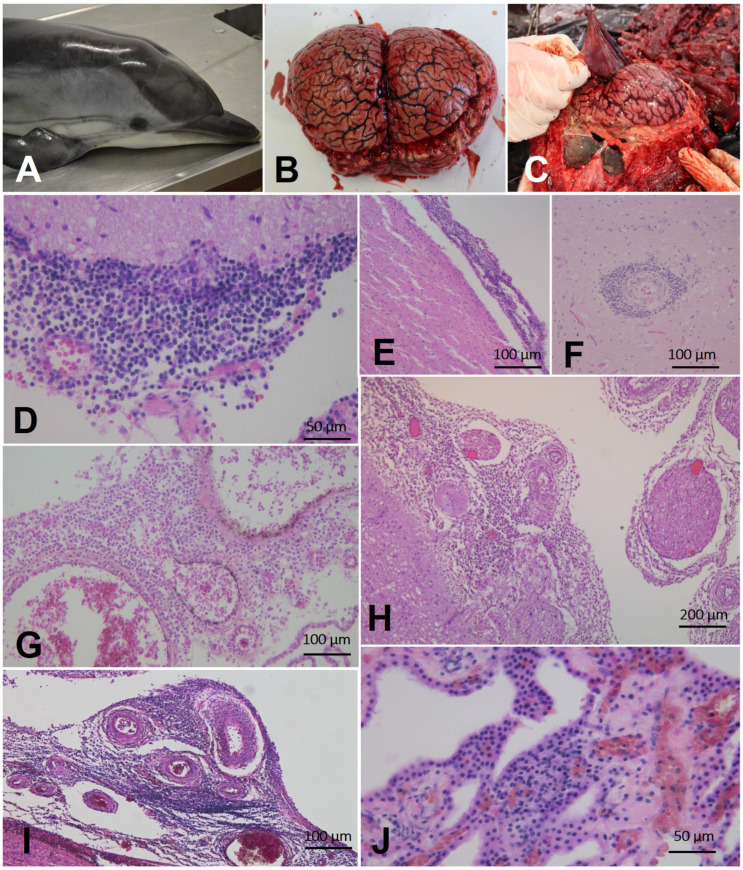
Gross and histological findings in striped dolphins (*Stenella coeruleoalba*) with neurobrucellosis. (**A**) Case 6. Dead stranded striped dolphin, presenting poor body condition. (**B**) Case 22. Hyperemic meninges over the cerebrum and cerebellum. (**C**) Case 24. Hyperemic meninges over the cerebrum. (**D**) Case 2. Non-suppurative meningitis. Cerebellar meninges are infiltrated by mononuclear cells 40x×. H&E. (**E**) Case 8. Non-suppurative meningitis. Cerebral cortex meninges are infiltrated by mononuclear cells. 10x×. H&E. (**F**) Case 7. Perivascular cuff characterized by the presence of lympho-monocytic cells. 20x×. H&E. **(G)** Case 5. Non-suppurative lympho-monocytic plexochoroiditis. 20x×. H&E. (**H**) Case 15. Severe non-suppurative lympho-monocytic meningitis, associated with non-suppurative periganglioneuritis 10x×. H&E. (**I**) Case 5. Severe non-suppurative meningitis. Meninges at the level of *medulla oblongata* are infiltrated by lympho-monocytic cells. 10x×. H&E. (**J**) Case 6. Non-suppurative lympho-monocytic plexo-choroiditis. 40x×. H&E.

Non-suppurative meningitis was detected in twenty animals, which was severe in five (Table 3; Supplementary Table S1; Figure 2G–H); non-suppurative encephalitis was detected in fourteen animals, being severe in four; non-suppurative plexus choroiditis was seen in ten animals, being severe in one (Table 3; Supplementary Table S1); non-suppurative cerebellitis (seven animals), myelitis (five animals), and polyradiculoneuritis (two animals) were the main lesions observed in other regions (Table 3; Supplementary Table S1; Figure 2G).

Associated lesions included the detection of protozoan cysts in Cases 3 and 6 (Table 3; Supplementary Table S1).

Pathological changes were also detected in three animals (Cases 11, 12, 13), despite their negativity for *Brucella* at CNS level. In detail, in Case 3, a multifocal acute encephalitis of unknown origin, associated with microgranulomas and perivascular hemorrhages, was evident; in Case 6, a non-suppurative meningoencephalitis, associated with mononuclear cell perivascular cuffs, vasculitis, and gliosis, alongside the presence of widespread *T. gondii* IHC-positive protozoan cysts of distinct size, was observed. Case 12 exhibited a severe chronic monocytic meningitis of unknown origin

##### Microscopic *Brucella ceti*-Associated Findings (Tissues Other Than CNS)

A minority of animals with neurobrucellosis (8/20) also showed *B. ceti*-associated lesions in other tissues (Cases 3, 6, 10, 16, 17, 18, 19, 21) (Supplementary Material Tables S1 and S2).

Overall, considering all the 23 animals with histopathological findings, *B. ceti-*associated lesions in positive tissues other than CNS involved splenitis (and/or lymphoid necrosis) (4/14; 20%), lymphadenitis (and/or lymphoid necrosis) (4/12; 33.3%), reproductive tract inflammation (4/12; 33.3%), myocarditis (and/or necrosis) (2/11; 18.1%), pneumonia (3/19; 15.7%), necrotizing hepatitis (2/15; 13.3%), and mastitis (1/3; 33.3%) (Table 2). The lesions affecting lymphoid tissues included splenitis and generalized lymphoid necrosis (spleen, prescapular, and pulmonary lymph nodes) (Case 6), splenitis, with infiltration of monocyte-macrophage type cells, and hyperplastic lymphadenitis in pancreatic and parotid lymph nodes (Case 10), lympho-histiocytic splenitis (Case 16), reactive lymphadenitis associated with congestion (Case 17), reactive lymphadenitis in prescapular lymph nodes (Case 19), and lymphoid hyperplasia in spleen (Case 21).

The lesions in reproductive organs were represented by lympho-monocytic oophoritis and lympho-monocytic endometritis (Case 10), lymphocytic orchitis (Case 12), endometritis with monocytic-macrophagic infiltration (Case 16), and severe non-suppurative endometritis (Case 18).

Lesions in the cardiovascular and respiratory systems included focal myocardial necrosis, associated with edema and mild inflammatory infiltrate (Case 18), alongside a mononuclear interstitial myocarditis (Case 19), pneumonia associated with perivascular lymphocytic infiltrates (Case 3), broncho-interstitial pneumonia (Case 6), and a non-suppurative interstitial pneumonia with exudative alveolitis, emphysema, and bronchial calcifications (Case 10).

Lesions in other organs were represented by multifocal necrotizing hepatitis (Case 6), necrotizing hemorrhagic hepatitis (Case 18), and mastitis (Case 10).

A selected collection of the gross and microscopic features of *B. ceti*-associated CNS lesions in some of the animals under study are shown in Figure 2.

#### 3.1.3. Coinfections

Fifteen animals (15/24; 62.5%) were found to be coinfected with other pathogens. PCR assays confirmed co-infections with CeMV alone (8/15), with CeMV and *T. gondii* (2/15), with *T. gondii* alone (2/15), with CeMV, *T. gondii*, and HV (1/15), with CeMV and HV (1/15), and with HV (1/15) alone.

By means of IHC, Morbillivirus infection was additionally confirmed in Case 6, while *T. gondii* infection was recognized in Cases 6, 12, and 21. Moreover, protozoan cysts were detected at brain level in 3/5 *T. gondii*—molecularly positive animals (Cases 3, 6, 12) with anti-*T. gondii* and anti-Morbillivirus antibodies being also found in Cases 3 and 6, and Cases 6 and 12, respectively.

Supplementary Table S1 summarizes the results obtained by IHC, molecular, serological, and histological investigations.

#### 3.1.4. Cause of Death and Pathological Evaluation

Hypotheses on the cause(s) of death were formulated for almost all animals (23/24). For Case 4, considering the limited data available, the cause of death was undetermined (ND). In all the animals evaluated (23/23; 100%) the cause of death was associated with natural origin, specifically represented by infectious diseases.

In most of the animals investigated (14/23; 60.8%) and specifically, in Cases 1, 2, 5, 8, 14, 15, 16, 17, 18, 19, 20, 22, 23, and 24, the lesions seen at CNS level were consistent with *B. ceti* infection, and their stranding could have resulted from a severe cerebral impairment exclusively due to *B. ceti* infection. Interestingly, a strong suspect of a feeding behavioral alteration (pica) was raised for Case 16, which showed an evident dislocation of the laryngeal beak due to a foreign body ingestion, with subsequent difficulties in food intake.

In Cases 7, 9, and 10, CNS lesions were consistent with *B. ceti* infection, and the stranding could have resulted from a severe cerebral impairment associated with CeMV coinfection, while in Cases 3 and 6, a *T. gondii* coinfection, and in Case 21, a *T. gondii* and HV coinfection, could have led to the dolphins’ stranding.

In Cases 11 and 12, the role of *B. ceti* infection remains unknown. In the first case, the lesions in the spleen were not consistent with *B. ceti* infection and the stranding could have resulted from a severe cerebral impairment associated with brain inflammation of unknown origin. In the second case, the testicular lesions were consistent with a localized *B. ceti* infection, and the stranding could have resulted from a severe cerebral impairment due to a brain inflammation by *T. gondii*.

In Case 13, while the CNS lesions were of suspect *B. ceti* origin, a confirmation by isolation and/or PCR was not obtained, so the role of *B. ceti* infection in the dolphin’s stranding remains unknown.

Supplementary Table S1 includes the results of the evaluation of the hypotheses on the cause(s) of death and on the pathogenic role of *B. ceti* for each case considered, summarized in column “comments/COD”.

### 3.2. Brucella Infection Diagnosis

All the results are summarized in Supplementary Table S1.

The isolation of *B. ceti* was attempted and obtained in all 24 animals under investigations.

*B. ceti* was isolated from the CNS of 20/24 striped dolphins (83.3%) (Cases 1, 2, 3, 4, 5, 6, 7, 8, 9, 10, 14,15, 16, 17, 18, 19, 20, 22, 23, 24), including the isolation from CSF of Case 16, and, in most of these animals, CNS was the only positive tissue (13/20). In the remaining seven animals (Cases 1,3, 6, 10, 17, 18, 19) the isolation was obtained from at least one, to a maximum of twelve, types of tissue tested.

In four animals (Cases 11, 12, 13, 21) *B. ceti* was isolated only from the spleen (Cases 11 and 21), ovary (Case 13), and testicle (Case 12).

The PCR for *Brucella* spp. detection directly from tissues was performed in 16/24 animals (66.6%) (Cases 6, 7, 8, 9, 10, 11, 12, 13, 14, 15, 16, 17, 20, 21, 22, 24), with positive results obtained from CNS and/or other tissues of 12 animals (12/16; 75%) (Cases 6, 7, 9, 19, 11, 12, 15, 16, 17, 20, 21, 24), and with evidence of a systemic spread in two cases (Cases 6 and 10). Notably, in Case 10, *Brucella* spp. was detected in a sampled lungworm (fam. Pseudaliidae).

The RBT test was performed using the blood serum of eleven animals (11/24; 45.8%)*,* and the test result was positive in six (6/11; 54.5%) (Cases 9, 10, 18, 19, 20, 23) and negative in the remaining five (Cases 6, 7, 12, 14, 15) samples.

### 3.3. Brucella ceti Epidemiological Data

**Figure 3:**
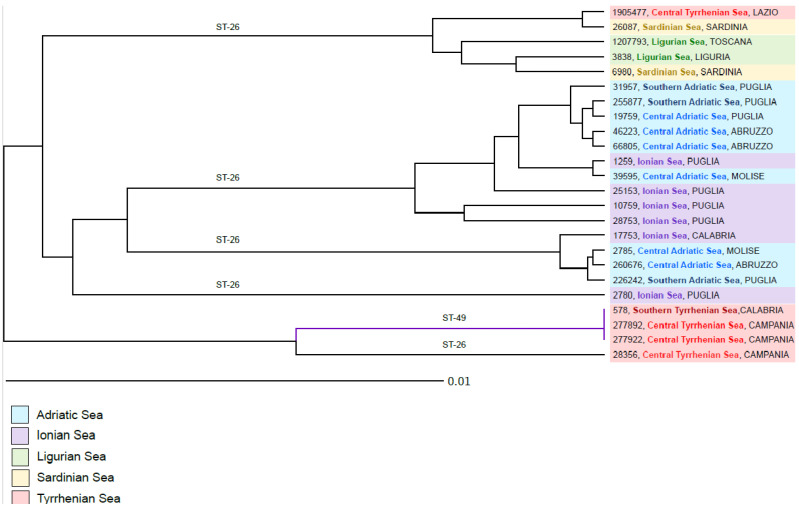
UPGMA tree generated using cgMLST for 24 strains of *B. ceti* isolated from striped dolphins stranded along the Italian coastline. The tree was calculated by pairwise comparison of 2076 core genes with missing values ignored. The origin of the stranded dolphins is highlighted with distinct colors and the MLST profiles are shown on the tree branches.

The genotyping of *B. ceti* using MLST classified 21/24 strains as sequence type 26 (ST-26) and 3/24 as sequence type 49 (ST-49). The analysis using cgMLST allowed us to examine the genetic relationship between the 24 strains included in our study. The strains were divided into four different clades (Clade 1: strains 1905477, 26087, 1207793, 3838, and 6980; Clade 2: strains 31957, 255877, 19759, 46223, 66805, 1259, 39595, 25153, 10759, and 28753; Clade 3: 17753, 2785, 260676, and 226242; Clade 4: 578, 277892, 277922, and 28356) and one branch containing a single strain (2780) (Figure 3). The maximum distance between the genomes was 61 core genes and the distance between the clades ranged between 37 and 44 core genes. Clade 1 was composed of strains isolated from dolphins found stranded on the western coasts of Italy, including the Central Tyrrhenian, Sardinian, and Ligurian Seas. The maximum allele distance within the clade reached 18; interestingly, however, two genomes from the Tyrrhenian Sea and the Sardinian Sea, 1905477 and 26087, were very closely genetically related (only two alleles of difference). Both Clade 2 and Clade 3 contained strains isolated along the eastern coastlines of the Adriatic and the Ionian Sea. The genetic distances between the strains in Clade 2 ranged from 1 to 20 alleles, while within Clade 3 we detected a maximum distance of only 5 core genes. The genetic variation between the genomes in Clade 2 was likely influenced not only by the geographic location of dolphins’ habitat, but also by the date of strain isolation, which ranged from 2012 to 2020. Clade 4, composed of strains isolated from dolphins stranded along the coastline of the Central and Southern Tyrrhenian Sea, was further divided into two branches, one containing three genetically identical strains assigned to ST-49 isolated in 2019 and 2021, and one isolate of ST26 located 28 alleles away from the other three strains.

**Figure 4:**
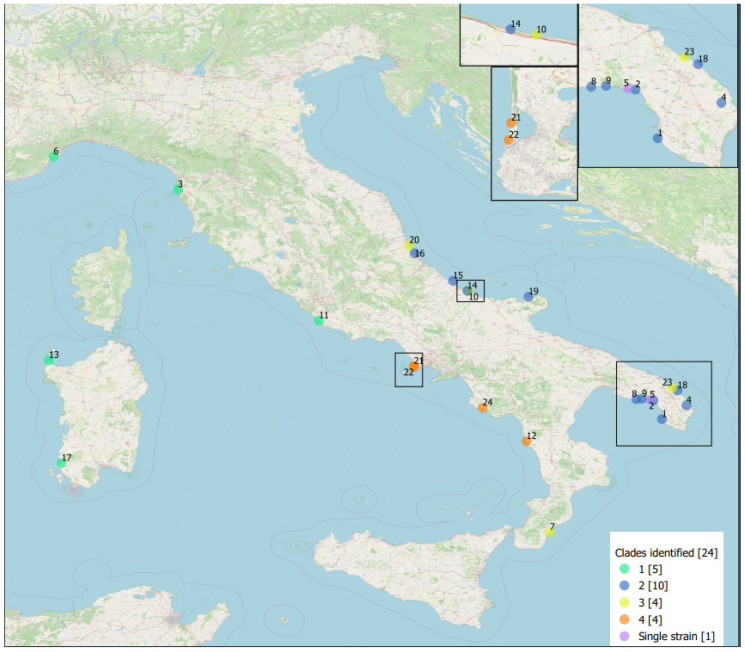
Stranding sites of striped dolphins infected by *B. ceti* under study, Italy, 2012–2021, by clade. Geographical mapping was obtained by QGIS software using the geographical coordinates found from the strandings (source monitoring of cetacean strandings on Italian coasts, http://mammiferimarini.unipv.it/index_en.php, accessed on 18 May 2023). The square brackets in the legend represent the overall number of strains and the number of strains for each clade identified. Each dot represents a stranding and is identified by the Case Id number of Supplementary Table S1. The Italy map was used under a CC BY-SA copyright from OpenStreetMap contributors (https://www.openstreetmap.org/copyright/en, accessed on 18 May 2023).

Plotting these clades on the geographic map suggests a link between their phylogeny and topographical distribution (Figure 4).

### 3.4. Statistical Analyses

Neither the univariate nor the multivariate analysis showed any statistically significant differences between the ST26 and ST49 *B. ceti* strains recovered from the investigated striped dolphins.

## 4. Discussion

This study represents an exhaustive survey of *B. ceti* infection in cetaceans from Italian waters, which included a detailed examination of 24 cases diagnosed between 2012 and 2021, along with a complete characterization of the *B. ceti* isolates. In comparison with the results of the first survey performed on eight animals [36], here we documented the circulation of a new sequence type (ST 49), and the occurrence of *B. ceti* infection extended to almost all marine sectors, instead of geographically restricted areas.

We isolated *B. ceti* from the CNS of most of the striped dolphins under investigation (83.3%), while, in a few animals (*N* = 4), the isolation was obtained only from lymphoid tissues and reproductive tract organs.

In seven animals, besides the CNS, *B. ceti* was isolated from other tissues, with two dolphins also showing clear evidence of severe systemic infection (Cases 10 and 18).

Although we observed several pathologic changes typically not consistent with *B. ceti* infection, we detected peculiar gross lesions associated with *Brucella* infection, mostly represented by hyperemic meninges and/or brain, which were observed in 17 animals [9,10,16,17]. Other *B. ceti*-associated findings were detected in the reproductive organs of four adult females, which were affected by endometritis (Cases 10, 16, 17, 19). Another feature observed was the increased volume or the hemorrhagic CSF appearance (Cases 10, 18, 20).

In our study, histological analyses revealed a strong correlation between *B. ceti* infection and neuropathological findings of different severity and frequency, which was in accordance with the previous descriptions [16,71]. Neurobrucellosis was indeed observed in most of the animals investigated (20/23), also associated, in a minority of cases (8/20), with *B. ceti*-associated lesions in other tissues (Cases 3, 6, 10, 16, 17, 18, 19, 21), including splenitis and/or generalized lymphoid necrosis, reactive lymphadenitis, necrotizing hepatitis, myocardial necrosis and myocarditis, reproductive tract inflammation, mastitis, and interstitial pneumonia.

The majority of *B. ceti-*positive animals displayed severe non-suppurative meningitis, along with encephalitis, choroiditis, cerebellitis, and myelitis. Specifically, non-suppurative meningoencephalitis, meningitis, or meningoencephalomyelitis, which were detected in the CNS of 20 *B. ceti-*infected animals, were similar to those reported in neurobrucellosis-affected humans [32,72], as well as in neurobrucellosis-affected striped dolphins elsewhere [9,12,16,17,18,33,36,73]. Notably, a relationship between a specific pulmonary *B. ceti*-associated lesion (interstitial pneumonia) and a severe parasitic bronchopneumonia by nematodes (from which *Brucella ceti* was isolated) was demonstrated in Case 10.

We detected co-infections in more than half of the animals investigated, mainly involving CeMV and *T. gondii*, as previously reported in several cetacean species infected by *Brucella* spp. and CeMV [21,71,74] or *T. gondii* [18,23]. Moreover, co-infection with Herpesvirus (HV) was also detected in three cases [71,75].

A potentially relevant role played by CeMV in initiating the animal’s decline could be suggested in 3/12 cases of co-infection with the virus (Cases 6, 10, and 19), along with specific immunopositivity and the presence of antibodies in Case 6, with evidence of systemic spread of *B. ceti* infection and the presence of several lesions consistent with this bacterial infection.

Likewise, the signs of *T. gondii* infection were observed in 4/5 cases of co-infection, and the presence of protozoan cysts at the cerebral level, the specific immunopositivity, and the presence of antibodies may also support the potentially relevant role played by *T. gondii* in initiating the animals’ decline.

A potential pathogenic role could have also been played by Herpesvirus in one of the cases of co-infection detected, specifically in Case 21, where classical CNS lesions associated with the presence of alphaherpesviruses [19,71,75,76,77,78], represented by neuronal necrosis, were detected. On the other hand, the absence of HV antigens in the CNS of the other two cases (Cases 17 and 22), and the absence of specific lesions in HV-positive tissues or the evidence of systemic infections [79,80,81], make the role of HV infection uncertain. Its involvement in the host’s immune response impairment could not, however, be negligible, as seen in the previous reports of CeMV [80,82].

Meningitis and encephalitis, associated with a wide range of pathogens (viral, bacterial, protozoan, parasitic), are among the leading known natural causes of death in stranded cetaceans [71]. Considering the pathogenic role of *B. ceti* in the striped dolphins under study, for most of the animals investigated by means of in-depth anatomo-histopathological analyses (14/23), the stranding could have resulted from a severe cerebral impairment associated exclusively with *B. ceti* infection. For the additional six cases, the stranding could have resulted from a severe cerebral impairment associated with a co-infection, specifically with CeMV (3/6), *T. gondii* (2/6), and *T. gondii* and HV together (1/6).

The role of *B. ceti* infection could not be determined in three cases, characterized by single isolation in spleen without associated pathological lesions (Case 11), single isolation at the testicular level with associated pathological lesions (Case 12), and single isolation at the ovary level, with microscopic features not available (Case 13).

Moreover, in Cases 2 and 10, the finding of lymphoid depletion, described before in dolphins with brucellosis [17,26], suggests an immunocompromised host response. Case 10 provide evidence of a CeMV co-infection and the presence of numerous *Brucella*-type lesions, involving spleen, lymph nodes, lung, ovarium, uterus, and mammary gland, representative of a severe systemic spread of *B. ceti* infection in the presence of anti-*Brucella* spp. antibodies.

Anti-*Brucella* spp. antibodies were detected in more than half of the eleven positive animals tested, and in three of them (Cases 10, 18, 19), they were associated with a severe systemic infection, characterized by *B. ceti* isolation and antigen detection in multiple tissues, alongside many *B. ceti*-associated lesions; in the three other dolphins (Cases 9, 20, 23), the infection was detected only at CNS level. Negative results were mostly associated with a single involvement of CNS (Cases 7, 12, 14, 15), and only in Case 6 were they associated with a severe systemic infection. This supports the evidence that the use of *B. abortus* antigen may lead to false-negative results, and other serological tests, such as the ELISA test or RBT test with *B. ceti* antigen, could be used as complementary methods to detect the serological response to *B. ceti* in dolphins, as previously shown [9,16,83]. Although the limited number of samples hampers any definitive conclusions on the use of RBT for the detection of *Brucella* spp. infection in dolphins, the positive results obtained in cases with a severe systemic infection in serum samples collected from animals having a good conservation status support a true positive result [9].

The highest frequency of *B. ceti* infection was confirmed in adults, and this observation seems in accordance with the results obtained in a previous study on marine mammals of the genus *Stenella* stranded in Brazil [26]. Additionally, more than a half of the animals showed a poor body condition, and almost all animals had empty stomachs at necropsy, suggesting an impairment in their foraging ability, likely caused by the disease and impairment of neurological functions [16,17,84].

Considering the stranding location of all striped dolphins under study, the circulation of *B. ceti* appears to extend to almost all Italian marine sectors, especially in the central-southern Adriatic and Ionian Seas.

None of the cases considered in this report stranded alive, so it was not possible to observe neurological symptoms at the time of stranding.

The MLST analysis of *B. ceti* isolated from stranded striped dolphins showed that most of the strains belonged to ST26, as previously described, and three strains were assigned to ST49. ST49, although not reported in the literature, has been previously associated with *B. ceti* strains isolated from dolphins in the United Kingdom and in Spain (https://pubmlst.org/, accessed on 18 May 2023). The cgMLST divided the Italian *B. ceti* population into four clades; however, the maximum distance between all the analyzed strains did not exceed 61 core genes, suggesting the presence of a recent common ancestor. Interestingly, within the clades, we identified closely related strains isolated in different years, suggesting that the genetic variation in *B. ceti* in the striped dolphin population in Italy may not be high. The division of the clades followed the geographic origin of the samples, with two clades containing the strains isolated from animals stranded along the eastern coastlines of Italy, and the two other clades composed of the strains from the western seas. We previously reported a strong phylogeographic segregation of the *B. ceti* isolates [36], and the results of the analysis of addition of 16 new genome sequences further support this observation. Here, we mapped the presence of the four clades in six distinct sea sectors, namely, Southern Adriatic, Central Adriatic, Ionian, Southern Tyrrhenian, Central Tyrrhenian, and Sardinian and Ligurian Seas. Again, the segregation was confirmed and the association with the dolphin population was also suggested. Further studies linking the distribution and behavior of striped dolphin pods in the European seas and the molecular evolution of *B. ceti* strains could provide more insights into the routes of transmission of *B. ceti* infection among the marine mammals.

In summary, our results provide novel data and pathological evidence of *B. ceti* infection in cetacean species in Italy and the geographic distribution range of this agent in Italian waters. Considering the results of this survey and the other data available [18,23,36], the occurrence of *B. ceti* infection in cetaceans stranded along the Italian coastline appears to be extended to almost all marine sectors and especially to the central-southern Adriatic and Ionian Seas.

The severity of *B. ceti*-associated lesions reported in the present study supports the role of *B. ceti* as a primary neurotropic pathogen in striped dolphins, as well as a probable cause of stranding events and death, as previously described elsewhere [21,34]. In this regard, our results corroborate previous reports indicating that striped dolphins are highly susceptible hosts for developing neurobrucellosis in comparison with the other cetaceans [17] and confirming neurobrucellosis as one of the most significant lesions’ patterns associated with *B. ceti* infection [17,18,21,23,85]. Additional studies are required to identify the mechanisms, as well as the pathogen- and the host-related factors, driving the neuro-invasion process in *B. ceti* infection and the role of specific virulence determinants in colonization and persistence in the host’s CNS [37].

No differences were seen in *B. ceti-*associated lesions and, furthermore, most of the cases (87%) clustered into ST26. With such small samples, statistical associations are very difficult to find.

To better understand the interactions between pathogen, host, and environmental factors, in close agreement with the “One Health” concept, a detailed understanding of the effects of pollutant-related immunotoxicity, suggested by some case reports [23,86], is additionally required, particularly in the light of the conflicting results obtained using ex vivo models [87].

Surveillance of cetacean strandings in Italy involves organizations from governmental and academic institutions with different areas of expertise such as public health, animal health, and environment. Such a network made this study possible, and our findings highlight the importance of maintaining a multidisciplinary and standardized approach in the monitoring of stranded cetaceans, with epidemiological and laboratory data truly shared across sectors.

Finally, based on the known zoonotic nature of *B. ceti* [88,89,90], it is strongly recommended to maintain proper handling of stranded animals and to adopt all necessary biosafety and biosecurity measures and protocols during post mortem and diagnostic investigations, to avoid the risk of the disease transmission to humans, as well as exposure to other neglected zoonoses.

## 5. Conclusions

Consistent with a previous survey [36], the present study confirms the role of *B. ceti* as a primary neurotropic pathogen for striped dolphins from Italian seas. Contrary to the idea that *B. ceti* infection is limited to specific areas, the circulation instead appears to extend to almost all Italian marine sectors, especially in the central-southern Adriatic and Ionian Seas. The genotyping of a higher number of strains allowed the identification of a new sequence type (ST49), in addition to the common, previously detected ST26, and the WGS provided novel data on the separation of the Italian *B. ceti* population into four clades. These data highlight the need for continuous surveillance and monitoring studies on stranded cetaceans, while maintaining a multidisciplinary and standardized approach, to improve knowledge of the impact and the evolution of this pathogen along the Italian coastline and in the Mediterranean Sea.

## Figures and Tables

**Table 1:** Overview of diagnostic investigations conducted per case. Cases are listed in chronological order.

Case ID	Histological	Immuno-Histochemical	Biomolecular			Serological		*Brucella* Infection Diagnosis		
			CeMV °	*T.* *gondii °°*	HV °°°	CeMV *	*T.* gondii*	Isolation and Identification **	PCR from Tissues	Serological (RBT) * and ***
1	x		x	x				X		
2	x		x	x				X		
3	x		x	x		x	x	X	X ^	
4			x	x				X		
5	x		x	x				X		
6	x	X (Morbillivirus; *T. gondii*) *	x	x	x	x		X	X ^	x
7	x		x	x		x	x	X	X ^	x
8	x		x	x				X	X ^^^	
9	x		x	x				X	X ^^^	x
10	x		x	x				X	X ^^	
11	x		x	x	x			X	X ^	
12	x	X (Morbillivirus; *T. gondii*) *	x	x		x		X	X ^	x
13	x		x	x				X	X ^	
14	x		x	x		x		X	X ^^	x
15	x		x	x		x	x	X	X ^^	x
16	x		x	x				X	X ^^	
17	x		x	x	x			X	X ^	
18	x		x	x				X		x
19	x		x	x				X		x
20	x		x	x				X	X ^^	x
21	x	X (Morbillivirus; *T. gondii*) *	x	x	x			X	X ^	
22	x	X (Morbillivirus) *	x	x	x			X	X ^	
23	x		x	x				X		x
24	x		x	x	x			x	X ^^	

Legend: x: performed; *: Di Guardo et al., 2010; °: Verna et al., 2018; °°: Vitale et al., 2013; °°°: VanDevanter et al., 1996; **: OIE Manual, 2018; Alton 1988; Cloeckaert et al., 1995; ^: Baily et al., 1992; ^^: Bounaadja et al., 2009; ^^^: López-Goñi et al., 2008 and Kang et al., 2011; ***: Hernandez-Mora et al., 2008.

**Table 2:** *B. ceti*-associated lesions (gross/microscopic) in tissues/fluids from microbiologically and/or molecularly *B. ceti-*positive striped dolphins under investigation. Note that one animal was not considered (gross and microscopic data not available).

Gross Lesions	Striped Dolphins Examined
Hyperemic meninges and/or brain	17 of 23 (73.9%)
Reproductive tract inflammation	4 of 23 (17.3%)
Hemorrhagic cerebrospinal fluid/increased volume	3 of 8 (37.5%)
**Microscopic lesions**	
Non-suppurative encephalitis (±meningitis/myelitis)	20 of 23 (86.9%)
Splenitis (±necrosis)	4 of 14 (20%)
Hepatitis	2 of 15 (13.3%)
Myocarditis (±necrosis)	2 of 11 (18.1%)
Reproductive tract inflammation	4 of 12 (33.3%)
Lymphadenitis (±necrosis)	4 of 14 (33.3%)
Pneumonia	3 of 19 (15.7%)
Mastitis	1 of 3 (33.3%)

**Table 3:** *Brucella ceti*-associated lesions in the CNS from *Brucella-*positive striped dolphins.

Case ID	Cerebrum			Other Nervous System Regions Different from the Cerebrum	Associated Lesions Related to Other Pathogens	Co-Infections (CNS)
	**M**	**E**	**Pl-C**			
1	**+**	-	-	-	-	-
2	**+**	-	-	NS cerebellitis		-
3	+ *	**+**	-	-	scattered protozoan cysts with a few granulomatous foci	*T. gondii*
5	+ *	+ *	**+**	NS inflammation in medulla oblongata *		
6	**+**	**+**	**+**	granulomatous cerebellitis, with malacia and protozoan cysts	protozoan cysts	CeMV— *T. gondii*
7	**+**	**+**	-	-	-	CeMV
8	+ *	+ *	**+**	NS myelitis	-	-
9	**+**	**+**	-	NS cerebellitis and myelitis	-	CeMV
10	**+**	-	-	NS cerebellitis, NS perineuritis and periganglioneuritis at spinal cord cervical level		CeMV
14	**+**	+ *	**+**	NS cerebellitis, myelitis.		-
15	+ *	-	-	NS inflammation in medulla oblongata and perineuritis		-
16	**+**	-	-	NS inflammation in medulla oblongata		
17	+ *	-	-	**-**	-	**-**
18	**+**	**+**	**+**	NS cerebellitis, inflammation in medulla oblongata	-	
19	**+**	**+**	**+**	NS inflammation in medulla oblongata and myelitis	-	**-**
20	**+**	**+**	-	NS inflammation in medulla oblongata and myelitis	-	**-**
21	**+**	**+**	**+**			*T. gondii*-HV
22	**+**	+ *	**+**		-	
23	**+**	**+**	**+**	NS cerebellitis	-	-
24	**+**	**+**	**+**		-	-

**Legend**: NA: sample not available for histopathology; NS: non suppurative; CNS: central nervous system; M: meningitis; E: encephalitis; Pl-C: plexus choroiditis; CeMV: Cetacean Morbillivirus; HV: Herpesvirus. *: severe.

## Data Availability

The WGS data is submitted at NCBI GenBank with the following accession number: PRJNA623338.

## Supplementary Materials

The following supporting information can be downloaded at: https://www.mdpi.com/article/10.3390/pathogens12081034/s1. Table S1: *Brucella ceti*-infected striped dolphins stranded and dead along the Italian coastline between 2012 and 2021, arranged in chronological order, with the corresponding ID strain; Table S2: Cases organized for the multivariate logistic regression model to evaluate associations between *B. ceti* ST and stranding area, age class, *B. ceti*-associated organ inflammation (histopathological and/or gross findings), and Morbillivirus infectious status.

## References

[1] Corbel M.J., World Health Organization (2006). Food and Agriculture Organization of the United Nations; International Office of Epizootics. Brucellosis in Humans and Animals.

[2] Whatmore A.M. (2009). Current Understanding of the Genetic Diversity of Brucella, an Expanding Genus of Zoonotic Pathogens. Infect. Genet. Evol..

[3] Godfroid J., Scholz H.C., Barbier T., Nicolas C., Wattiau P., Fretin D., Whatmore A.M., Cloeckaert A., Blasco J.M., Moriyon I. (2011). Brucellosis at the Animal/Ecosystem/Human Interface at the Beginning of the 21st Century. Prev. Vet. Med..

[4] Ewalt D.R., Payeur J.B., Martin B.M., Cummins D.R., Miller W.G. (1994). Characteristics of a *Brucella* Species from a Bottlenose Dolphin (*Tursiops Truncatus*). J. Vet. Diagn. Investig..

[5] Ross H., Foster G., Reid R., Jahans K., MacMillan A. (1994). Brucella Species Infection in Sea-Mammals. Vet. Rec..

[6] Foster G., Osterman B.S., Godfroid J., Jacques I., Cloeckaert A. (2007). *Brucella ceti* sp. Nov. and *Brucella pinnipedialis* sp. Nov. for Brucella Strains with Cetaceans and Seals as Their Preferred Hosts. Int. J. Syst. Evol. Microbiol..

[7] Whatmore A.M., Dawson C.E., Groussaud P., Koylass M.S., King A.C., Shankster S.J., Sohn A.H., Probert W.S., McDonald W.L. (2008). Marine Mammal Brucella Genotype Associated with Zoonotic Infection. Emerg. Infect. Dis..

[8] Nymo I.H., Tryland M., Godfroid J. (2011). A Review of Brucella Infection in Marine Mammals, with Special Emphasis on Brucella Pinnipedialis in the Hooded Seal (*Cystophora cristata*). Vet. Res..

[9] Guzmán-Verri C., González-Barrientos R., Hernández-Mora G., Morales J.A., Baquero-Calvo E., Chaves-Olarte E., Moreno E. (2012). Brucella Ceti and Brucellosis in Cetaceans. Front. Cell Infect. Microbiol..

[10] Hernández-Mora G., Palacios-Alfaro J.D., González-Barrientos R. (2013). Wild Reservoirs of Brucellosis: Brucella in Aquatic Environments. Rev. Sci. Tech. De. L’oie.

[11] Whatmore A.M., Dawson C., Muchowski J., Perrett L.L., Stubberfield E., Koylass M., Foster G., Davison N.J., Quance C., Sidor I.F. (2017). Characterisation of North American Brucella Isolates from Marine Mammals. PLoS ONE.

[12] González L., Patterson I.A., Reid R.J., Foster G., Barberán M., Blasco J.M., Kennedy S., Howie F.E., Godfroid J., MacMillan A.P. (2002). Chronic Meningoencephalitis Associated with *Brucella* Sp. Infection in Live-Stranded Striped Dolphins (*Stenella coeruleoalba*). J. Comp. Pathol..

[13] Miller W.G., Adams L.G., Ficht T.A., Cheville N.F., Payeur J.P., Harley D.R., House C., Ridgway S.H. (1999). Brucella-Induced Abortions, and Infection in Bottlenose Dolphins (*Tursiops truncatus*). J. Zoo. Wildl. Med..

[14] Foster G., MacMillan A.P., Godfroid J., Howie F., Ross H.M., Cloeckaert A., Reid R.J., Brew S., Patterson I.A.P. (2002). A Review of *Brucella* Sp. Infection of Sea Mammals with Particular Emphasis on Isolates from Scotland. Vet. Microbiol..

[15] Ohishi K., Zenitani R., Bando T., Goto Y., Uchida K., Maruyama T., Yamamoto S., Miyazaki N., Fujise Y. (2003). Pathological and Serological Evidence of Brucella-Infection in Baleen Whales (Mysticeti) in the Western North Pacific. Comp. Immunol. Microbiol. Infect. Dis..

[16] Hernández-Mora G., González-Barrientos R., Morales J.-A., Chaves-Olarte E., Guzmán-Verri C., Baquero-Calvo E., De-Miguel M.-J., Marín C.-M., Blasco J.-M., Moreno E. (2008). Neurobrucellosis in Stranded Dolphins, Costa Rica. Emerg. Infect. Dis..

[17] González-Barrientos R., Morales J.-A., Hernández-Mora G., Barquero-Calvo E., Guzmán-Verri C., Chaves-Olarte E., Moreno E. (2010). Pathology of Striped Dolphins (*Stenella coeruleoalba*) Infected with Brucella Ceti. J. Comp. Pathol..

[18] Alba P., Terracciano G., Franco A., Lorenzetti S., Cocumelli C., Fichi G., Eleni C., Zygmunt M.S., Cloeckaert A., Battisti A. (2013). The Presence of Brucella Ceti ST26 in a Striped Dolphin (*Stenella coeruleoalba*) with Meningoencephalitis from the Mediterranean Sea. Vet. Microbiol..

[19] Sierra E., Sánchez S., Saliki J.T., Blas-Machado U., Arbelo M., Zucca D., Fernández A. (2014). Retrospective Study of Etiologic Agents Associated with Nonsuppurative Meningoencephalitis in Stranded Cetaceans in the Canary Islands. J. Clin. Microbiol..

[20] Olsen S.C., Palmer M.V. (2014). Advancement of Knowledge of Brucella Over the Past 50 Years. Vet. Pathol..

[21] Isidoro-Ayza M., Ruiz-Villalobos N., Pérez L., Guzmán-Verri C., Muñoz P.M., Alegre F., Barberán M., Chacón-Díaz C., Chaves-Olarte E., González-Barrientos R. (2014). Brucella Ceti infection in Dolphins from the Western Mediterranean Sea. BMC Vet. Res..

[22] Colegrove K., Venn-Watson S., Litz J., Kinsel M., Terio K., Fougeres E., Ewing R., Pabst D., McLellan W., Raverty S. (2016). Fetal Distress and in Utero Pneumonia in Perinatal Dolphins during the Northern Gulf of Mexico Unusual Mortality Event. Dis. Aquat. Organ..

[23] Grattarola C., Giorda F., Iulini B., Pintore M.D., Pautasso A., Zoppi S., Goria M., Romano A., Peletto S., Varello K. (2016). Meningoencephalitis and *Listeria monocytogenes*, *Toxoplasma gondii* and *Brucella* Spp. Coinfection in a Dolphin in Italy. Dis. Aquat. Organ..

[24] Davison N.J., Perrett L.L., Dawson C., Dagleish M.P., Haskins G., Muchowski J., Whatmore A.M. (2017). *Brucella ceti* Infection in a Common Minke Whale (*Balaenoptera acutorostrata*) with Associated Pathology. J. Wildl. Dis..

[25] Buckle K., Roe W.D., Howe L., Michael S., Duignan P.J., Burrows E., Ha H.J., Humphrey S., McDonald W.L. (2017). Brucellosis in Endangered Hector’s Dolphins *(Cephalorhynchus hectori)*. Vet. Pathol..

[26] Sánchez-Sarmiento A.M., Carvalho V.L., Díaz-Delgado J., Ressio R.A., Fernandes N.C.C.A., Guerra J.M., Sacristán C., Groch K.R., Silvestre-Perez N., Ferreira-Machado E. (2019). Molecular, Serological, Pathological, Immunohistochemical and Microbiological Investigation of *Brucella* Spp. in Marine Mammals of Brazil Reveals New Cetacean Hosts. Transbound. Emerg. Dis..

[27] Sierra A., Paolicelli R.C., Kettenmann H. (2019). Cien Años de Microglía: Milestones in a Century of Microglial Research. Trends Neurosci..

[28] Davison N., Dagleish M., Ten Doeschate M., Muchowski J., Perrett L., Rocchi M., Whatmore A., Brownlow A. (2021). Meningoencephalitis in a Common Minke Whale Balaenoptera Acutorostrata Associated with Brucella Pinnipedialis and Gamma-Herpesvirus Infection. Dis. Aquat. Organ..

[29] Curtiss J.B., Colegrove K.M., Dianis A., Kinsel M.J., Ahmed N., Fauquier D., Rowles T., Niemeyer M., Rotstein D.S., Maddox C.W. (2022). Brucella Ceti Sequence Type 23, 26, and 27 Infections in North American Cetaceans. Dis. Aquat. Organ..

[30] Granados-Zapata A., Robles-Malagamba M.J., González-Barrientos R., Kot B.C.-W., Barquero-Calvo E., Cordero-Chavaría M., Suárez-Esquivel M., Guzmán-Verri C., Palacios-Alfaro J.D., Tien-Sung C. (2022). Pathological Studies and Postmortem Computed Tomography of Dolphins with Meningoencephalomyelitis and Osteoarthritis Caused by *Brucella ceti*. Oceans.

[31] Di Francesco G., Petrini A., D’angelo A.R., Di Renzo L., Luciani M., Di Febo T., Ruggieri E., Petrella A., Grattarola C., Iulini B. (2019). Immunohistochemical Investigations on Brucella Ceti-Infected, Neurobrucellosis-Affected Striped Dolphins (*Stenella coeruleoalba*). Vet. Ital..

[32] Obiako O.R., Ogoina D., Danbauchi S.S., Kwaifa S.I., Chom N.D., Nwokorie E. (2010). Neurobrucellosis—A Case Report and Review of Literature. Niger. J. Clin. Pract..

[33] Bossart G.D. (2011). Marine Mammals as Sentinel Species for Oceans and Human Health. Vet. Pathol..

[34] Davison N.J., Cranwell M.P., Perrett L.L., Dawson C.E., Stubberfield E.J., Deaville R., Jepson P.D., Jarvis D.S. (2009). Meningoencephalitis Associated with *Brucella* Species in a Live-Stranded Striped Dolphin (*Stenella coeruleoalba*) in South-West England. Vet. Rec..

[35] Di Francesco G., Di Renzo L., Garofolo G., Tittarelli M., Di Guardo G. (2020). Two Neurotropic Pathogens of Concern for Striped Dolphins. Vet. Rec..

[36] Garofolo G., Petrella A., Lucifora G., Di Francesco G., Di Guardo G., Pautasso A., Iulini B., Varello K., Giorda F., Goria M. (2020). Occurrence of Brucella Ceti in Striped Dolphins from Italian Seas. PLoS ONE.

[37] Di Guardo G., Centelleghe C., Mazzariol S. (2018). Cetacean Host-Pathogen Interaction(s): Critical Knowledge Gaps. Front. Immunol..

[38] Angelucci C.B., Giacominelli-Stuffler R., Baffoni M., Di Francesco C.E., Di Francesco G., Di Renzo L., Tittarelli M., Petrella A., Grattarola C., Mazzariol S. (2022). Cellular Prion Protein Expression in the Brain Tissue from *Brucella ceti*-Infected Striped Dolphins (*Stenella coeruleoalba*). Animals.

[39] Maquart M., Le Flèche P., Foster G., Tryland M., Ramisse F., Djønne B., Al Dahouk S., Jacques I., Neubauer H., Walravens K. (2009). MLVA-16 Typing of 295 Marine Mammal Brucella Isolates from Different Animal and Geographic Origins Identifies 7 Major Groups within *Brucella ceti* and *Brucella pinnipedialis*. BMC Microbiol..

[40] Suárez-Esquivel M., Baker K.S., Ruiz-Villalobos N., Hernández-Mora G., Barquero-Calvo E., González-Barrientos R., Castillo-Zeledón A., Jiménez-Rojas C., Chacón-Díaz C., Cloeckaert A. (2017). Brucella Genetic Variability in Wildlife Marine Mammals Populations Relates to Host Preference and Ocean Distribution. Genome Biol. Evol..

[41] Thompson L.A., Goertz C.E.C., Quakenbush L.T., Burek Huntington K., Suydam R.S., Stimmelmayr R., Romano T.A. (2022). Serological Detection of Marine Origin Brucella Exposure in Two Alaska Beluga Stocks. Animals.

[42] Garofolo G., Zilli K., Troiano P., Petrella A., Marotta F., Di Serafino G., Ancora M., Di Giannatale E. (2014). Brucella Ceti from Two Striped Dolphins Stranded on the Apulia Coastline, Italy. J. Med. Microbiol..

[43] Cvetnić Ž., Duvnjak S., Đuras M., Gomerčić T., Reil I., Zdelar-Tuk M., Špičić S. (2016). Evidence of Brucella Strain ST27 in Bottlenose Dolphin (*Tursiops truncatus*) in Europe. Vet. Microbiol..

[44] Duvnjak S., Špičić S., Kušar D., Papić B., Reil I., Zdelar-Tuk M., Pavlinec Ž., Đuras M., Gomerčić T., Hendriksen R.S. (2017). Whole-Genome Sequence of the First Sequence Type 27 Brucella Ceti Strain Isolated from European Waters. Genome Announc..

[45] Geraci J.R., Lounsbury V.J. (2005). Marine Mammals Ashore: A Field Guide for Strandings.

[46] Ijsseldijk L.L., Brownlow A.C., Mazzariol S. Best Practice on Cetacean Postmortem Investigation and Tissue Sampling Joint ACCOBAMS and ASCOBANS Document Editors. Proceedings of the 25th Meeting of the Advisory Committee.

[47] Carlini R. (2014). Biometric Measures Indicating Sexual Dimorphism in *Stenella Coeruleoalba* (Meyen, 1833) (Delphinidae) in the North-Central Tyrrhenian Sea. Aquat Mamm.

[48] Perrin W.F., Reilly S.B. (1984). Reproductive Parameters of Dolphins, and Small Whales of the Family Deiphinidae. Rep. Int. Whal. Comm..

[49] Cozzi B., Huggenberger S., Oelschläger H. (2017). Urinary System, Genital Systems, and Reproduction. Anatomy of Dolphins.

[50] Anderson R.M. (1978). The Regulation of Host Population Growth by Parasitic Species. Parasitology.

[51] Khalil L.F., Jones A., Bray R.A. (1995). Keys to the Cestode Parasites of Vertebrates. Trans. R. Soc. Trop. Med. Hyg..

[52] Pintore M.D., Mignone W., Di Guardo G., Mazzariol S., Ballardini M., Florio C.L., Goria M., Romano A., Caracappa S., Giorda F. (2018). Neuropathologic Findings in Cetaceans Stranded in Italy (2002–2014). J. Wildl. Dis..

[53] Di Guardo G., Proietto U., Di Francesco C.E., Marsilio F., Zaccaroni A., Scaravelli D., Mignone W., Garibaldi F., Kennedy S., Forster F. (2010). Cerebral Toxoplasmosis in Striped Dolphins (*Stenella coeruleoalba*) Stranded Along the Ligurian Sea Coast of Italy. Vet. Pathol..

[54] Verna F., Giorda F., Miceli I., Rizzo G., Pautasso A., Romano A., Iulini B., Pintore M.D., Mignone W., Grattarola C. (2017). Detection of Morbillivirus Infection by RT-PCR RFLP Analysis in Cetaceans and Carnivores. J. Virol. Methods.

[55] Vitale M. (2013). A Highly Sensitive Nested PCR for Toxoplasma Gondii Detection in Animal and Food Samples. J. Microb. Biochem. Technol..

[56] VanDevanter D.R., Warrener P., Bennett L., Schultz E.R., Coulter S., Garber R.L., Rose T.M. (1996). Detection and Analysis of Diverse Herpesviral Species by Consensus Primer PCR. J. Clin. Microbiol..

[57] OIE (2018). OIE Manual of Diagnostic Tests and Vaccines for Terrestrial Animals.

[58] Alton G.G., Jones L.M., Pietz D.E. (1988). Laboratory Techniques in Brucellosis.

[59] Cloeckaert A., Verger J.-M., Grayon M., Grepinet O. (1995). Restriction Site Polymorphism of the Genes Encoding the Major 25 KDa and 36 KDa Outer-Membrane Proteins of Brucella. Microbiology.

[60] Baily G.G., Krahn J.B., Drasar B.S., Stoker N.G. (1992). Detection of Brucella Melitensis and Brucella Abortus by DNA Amplification. J. Trop. Med. Hyg..

[61] Bounaadja L., Albert D., Chénais B., Hénault S., Zygmunt M.S., Poliak S., Garin-Bastuji B. (2009). Real-Time PCR for Identification of *Brucella* Spp.: A Comparative Study of IS711, Bcsp31 and per Target Genes. Vet. Microbiol..

[62] López-Goñi I., García-Yoldi D., Marín C.M., de Miguel M.J., Muñoz P.M., Blasco J.M., Jacques I., Grayon M., Cloeckaert A., Ferreira A.C. (2008). Evaluation of a Multiplex PCR Assay (Bruce-Ladder) for Molecular Typing of All *Brucella* Species, Including the Vaccine Strains. J. Clin. Microbiol..

[63] Kang S.-I., Her M., Kim J.W., Kim J.-Y., Ko K.Y., Ha Y.-M., Jung S.C. (2011). Advanced Multiplex PCR Assay for Differentiation of *Brucella* Species. Appl. Env. Microbiol..

[64] Andrews K.R., Karczmarski L., Au W.W.L., Rickards S.H., Vanderlip C.A., Bowen B.W., Gordon Grau E., Toonen R.J. (2010). Rolling Stones, and Stable Homes: Social Structure, Habitat Diversity and Population Genetics of the Hawaiian Spinner Dolphin (*Stenella longirostris*). Mol. Ecol..

[65] Bolger A.M., Lohse M., Usadel B. (2014). Trimmomatic: A Flexible Trimmer for Illumina Sequence Data. Bioinformatics.

[66] Bankevich A., Nurk S., Antipov D., Gurevich A.A., Dvorkin M., Kulikov A.S., Lesin V.M., Nikolenko S.I., Pham S., Prjibelski A.D. (2012). SPAdes: A New Genome Assembly Algorithm and Its Applications to Single-Cell Sequencing. J. Comput. Biol..

[67] Gurevich A., Saveliev V., Vyahhi N., Tesler G. (2013). QUAST: Quality Assessment Tool for Genome Assemblies. Bioinformatics.

[68] Jünemann S., Sedlazeck F.J., Prior K., Albersmeier A., John U., Kalinowski J., Mellmann A., Goesmann A., von Haeseler A., Stoye J. (2013). Updating Benchtop Sequencing Performance Comparison. Nat. Biotechnol..

[69] Arbelo M., Espinosa de los Monteros A., Herráez P., Andrada M., Sierra E., Rodríguez F., Jepson P., Fernández A. (2013). Pathology and Causes of Death of Stranded Cetaceans in the Canary Islands (1999–2005). Dis. Aquat. Organ..

[70] Giorda F., Ballardini M., Di Guardo G., Pintore M.D., Grattarola C., Iulini B., Mignone W., Goria M., Serracca L., Varello K. (2017). Postmortem Findings in Cetaceans Found Stranded in the Pelagos Sanctuary, Italy, 2007–2014. J Wildl Dis.

[71] Sierra E., Fernández A., Felipe-Jiménez I., Zucca D., Díaz-Delgado J., Puig-Lozano R., Câmara N., Consoli F., Díaz-Santana P., Suárez-Santana C. (2020). Histopathological Differential Diagnosis of Meningoencephalitis in Cetaceans: *Morbillivirus*, *Herpesviru*s, *Toxoplasma gondii*, *Brucella* sp., and *Nasitrema* sp. Front. Vet. Sci..

[72] Shakir R.A., Al-Din A.S.N., Araj G.F., Lulu A.R., Mousa A.R., Saadah M.A. (1987). Clinical Categories of Neurobrucellosis. Brain.

[73] Davison N.J., Brownlow A., Doeschate M.T., Dale E.-J., Foster G., Muchowski J., Perrett L.L., Rocchi M., Whatmore A.M., Dagleish M.P. (2021). Neurobrucellosis Due to Brucella Ceti ST26 in Three Sowerby’s Beaked Whales (*Mesoplodon bidens*). J. Comp. Pathol..

[74] West K.L., Levine G., Jacob J., Jensen B., Sanchez S., Colegrove K., Rotstein D. (2015). Coinfection and Vertical Transmission of *Brucella* and *morbillivirus* in a Neonatal Sperm Whale (*Physeter macrocephalus*) in Hawaii, USA. J. Wildl. Dis..

[75] Vargas-Castro I., Melero M., Crespo-Picazo J.L., de los Á Jiménez M., Sierra E., Rubio-Guerri C., Arbelo M., Fernández A., García-Párraga D., Sánchez-Vizcaíno J.M. (2021). Systematic Determination of Herpesvirus in Free-Ranging Cetaceans Stranded in the Western Mediterranean: Tissue Tropism and Associated Lesions. Viruses.

[76] Kennedy S., Lindstedt I.J., McAliskey M.M., McConnell S.A., McCullough S.J. (1992). Herpesviral Encephalitis in a Harbor Porpoise (*Phocoena phocoena*). J. Zoo. Wildl. Med..

[77] Esperón F., Fernández A., Sánchez-Vizcaíno J. (2008). Herpes Simplex-like Infection in a Bottlenose Dolphin Stranded in the Canary Islands. Dis. Aquat. Organ..

[78] van Elk C., van de Bildt M., van Run P., de Jong A., Getu S., Verjans G., Osterhaus A., Kuiken T. (2016). Central Nervous System Disease and Genital Disease in Harbor Porpoises (*Phocoena phocoena*) Are Associated with Different Herpesviruses. Vet. Res..

[79] Blanchard T.W., Santiago N.T., Lipscomb T.P., Garber R.L., McFee W.E., Knowles S. (2001). Two Novel Alphaherpesviruses Associated with Fatal Disseminated Infections in Atlantic Bottlenose Dolphins. J. Wildl. Dis..

[80] Arbelo M., Sierra E., Esperón F., Watanabe T., Bellière E., Espinosa de los Monteros A., Fernández A. (2010). Herpesvirus Infection with Severe Lymphoid Necrosis Affecting a Beaked Whale Stranded in the Canary Islands. Dis. Aquat. Organ..

[81] Soto S., González B., Willoughby K., Maley M., Olvera A., Kennedy S., Marco A., Domingo M. (2012). Systemic Herpesvirus and Morbillivirus Co-Infection in a Striped Dolphin (*Stenella coeruleoalba*). J. Comp. Pathol..

[82] Van Bressem M.-F., Duignan P., Banyard A., Barbieri M., Colegrove K., De Guise S., Di Guardo G., Dobson A., Domingo M., Fauquier D. (2014). Cetacean Morbillivirus: Current Knowledge and Future Directions. Viruses.

[83] Nymo I.H., Godfroid J., Åsbakk K., Larsen A.K., das Neves C.G., Rødven R., Tryland M. (2013). A Protein A/G Indirect Enzyme-Linked Immunosorbent Assay for the Detection of Anti-Brucella Antibodies in Arctic Wildlife. J. Vet. Diagn. Investig..

[84] Roca-Monge K., González-Barrientos R., Suárez-Esquivel M., Palacios-Alfaro J.D., Castro-Ramírez L., Jiménez-Soto M., Cordero-Chavarría M., García-Párraga D., Barratclough A., Moreno E. (2022). Age and Sexual Maturity Estimation of Stranded Striped Dolphins, Stenella Coeruleoalba, Infected with Brucella Ceti. Oceans.

[85] Muñoz P.M., Mick V., Sacchini L., Janowicz A., de Miguel M.J., Cherfa M.-A., Nevado C.R., Girault G., Andrés-Barranco S., Jay M. (2019). Phylogeography and Epidemiology of Brucella Suis Biovar 2 in Wildlife and Domestic Swine. Vet. Microbiol..

[86] Davison N.J., Perrett L.L., Law R.J., Dawson C.E., Stubberfield E.J., Monies R.J., Deaville R., Jepson P.D. (2011). Infection with *Brucella ceti* and High Levels of Polychlorinated Biphenyls in Bottlenose Dolphins (*Tursiops truncatus*) Stranded in South-West England. Vet. Rec..

[87] Nymo I.H., das Neves C.G., Tryland M., Bårdsen B.-J., Santos R.L., Turchetti A.P., Janczak A.M., Djønne B., Lie E., Berg V. (2014). Brucella Pinnipedialis Hooded Seal (*Cystophora cristata*) Strain in the Mouse Model with Concurrent Exposure to PCB 153. Comp. Immunol. Microbiol. Infect. Dis..

[88] Brew S.D., Perrett L.L., Stack J.A., MacMillan A.P., Staunton N.J. (1999). Human Exposure to Brucella Recovered from a Sea Mammal. Vet. Rec..

[89] Sohn A.H., Probert W.S., Glaser C.A., Gupta N., Bollen A.W., Wong J.D., Grace E.M., McDonald W.C. (2003). Human Neurobrucellosis with Intracerebral Granuloma Caused by a Marine Mammal *Brucella* Spp.. Emerg. Infect. Dis..

[90] McDonald W.L., Jamaludin R., Mackereth G., Hansen M., Humphrey S., Short P., Taylor T., Swingler J., Dawson C.E., Whatmore A.M. (2006). Characterization of a *Brucella* Sp. Strain as a Marine-Mammal Type despite Isolation from a Patient with Spinal Osteomyelitis in New Zealand. J. Clin. Microbiol..

