# Supplementary material for: Brucella ceti Infection in Striped Dolphins from Italian Seas: Associated Lesions and Epidemiological Data
Source: Pathogens. 2023 Aug 13;12(8):1034. doi: 10.3390/pathogens12081034 (PMC10459742; doi:10.3390/pathogens12081034)
Supplement: Supplementary file 1 [file pathogens-12-01034-s001.zip › Supplementary material Table S2.pdf]

Table S2. Cases organized for the multivariate logistic regression model to evaluate associations between *B. ceti* ST and stranding area, age class, *B. ceti*-associated organ inflammation (histopathological and/or gross findings) and Morbillivirus infectious status.

Case ID	sex	ST		Stranding area							Age class		*B. ceti*-associated organ inflammation								CeMV +
		26	49	I	L	CA	SA	CT	ST	S	juvenile	adult	Brain (CNS)	Repro.	LN	Spleen	Liver	Heart	Lung	Mammary gland	
1	M	**+**	-	**+**	-	-	-	-	-	-	**+**	-	**+**	NA	NA	NA	NA	NA	NA	na	-
2	F	**+**	-	**+**	-	-	-	-	-	-	**+**	-	**+**	NA	-	-	-	NA	NA	NA	-
3	M	**+**	-	-	**+**	-	-	-	-	-	-	**+**	**+**	-	-	NA	-	-	**+**	na	-
4	nd	**+**	-	-	-	-	**+**	-	-	-	nd	nd	NA	NA	NA	NA	NA	NA	NA	NA	-
5	M	**+**	-	**+**	-	-	-	-	-	-	**+**	-	**+**	NA	NA	NA	NA	NA	NA	na	-
6	M	**+**	-	-	**+**	-	-	-	-	-	**+**	-	**+**	NA	**+**	**+**	**+**	NA	**+**	na	**+**
7	F	**+**	-	**+**	-	-	-	-	-	-	-	**+**	**+**	NA	NA	NA	NA	NA	NA	NA	**+**
8	M	**+**	-	**+**	-	-	-	-	-	-	**+**	-	**+**	NA	NA	-	-	-	-	na	**+**
9	M	**+**	-	**+**	-	-	-	-	-	-	**+**	-	**+**	NA	NA	NA	NA	NA	-	na	**+**
10	F	**+**	-	-	-	**+**	-	-	-	-	-	**+**	**+**	**+**	**+**	**+**	NA	NA	**+**	**+**	**+**
11	F	**+**	-	-	-	-	-	**+**	-	-	**+**	-	-	NA	NA	-	-	-	-	NA	**+**
12	M	-	**+**	-	-	-	-	-	**+**	-	-	**+**	-	**+**	-	NA	NA	NA	-	na	**+**
13	F	**+**	-	-	-	-	-	-	-	**+**	-	+	-	NA	NA	NA	NA	NA	-	NA	**+**
14	F	**+**	-	-	-	**+**	-	-	-	-	-	**+**	**+**	-	-	-	NA	-	-	NA	-
15	F	**+**	-	-	-	**+**	-	-	-	-	**+**	-	**+**	-	-	-	-	NA	-	NA	-
16	F	**+**	-	-	-	**+**	-	-	-	-	-	**+**	**+**	**+**	-	**+**	-	NA	-	NA	-
17	F	**+**	-	-	-	-	-	-	-	**+**	-	**+**	**+**	**+**	**+**	NA	-	NA	-	NA	-
18	F	**+**	-	-	-	-	**+**	-	-	-	-	**+**	**+**	**+**	-	-	**+**	**+**	-	NA	-
19	F	**+**	-	-	-	**+**	-	-	-	-	-	**+**	**+**	NA	**+**	-	-	**+**	-	NA	**+**
20	F	**+**	-	-	-	**+**	-	-	-	-	-	**+**	**+**	-	-	-	-	-	-	-	-
21	M	-	**+**	-	-	-	-	**+**	-	-	-	**+**	**+**	-	NA	**+**	-	-	-	na	**+**
22	F	-	**+**	-	-	-	-	**+**	-	-	-	**+**	**+**	-	-	-	-	-	-	-	**+**
23	F	**+**	-	-	-	-	**+**	-	-	-	nd	nd	**+**	NA	NA	-	-	-	-	NA	-
24	F	**+**	-	-	-	-	-	-	**+**	-	-	**+**	**+**	-	-	NA	-	-	-	NA	**+**
**TOT**		**21**	**3**	**6**	**2**	**6**	**3**	**3**	**2**	**2**	**9/22**	**13/22**	**20/23**	**5/12**	**4/14**	**4/14**	**2/15**	**2/11**	**3/19**	**1/3**	**12/24**
**%**		**87,5**	**12,5**	**25**	**8**	**25**	**12,5**	**12,5**	**8**	**8**	**40.9**	**59,09**	**86,9**	**41,6**	**28,5**	**28,5**	**13,3**	**18,1**	**15,7**	**33,3**	**50**

NA: sample not available for histopathology; na: sample not available (male); not determined; I: Ionian Sea; L: Ligurian Sea; CA: Central Adriatic Sea; SA: Southern Adriatic Sea; CT: Central Tyrrhenian Sea; ST: Southern Tyrrhenian Sea; S: Sardinian Sea
